# Malaria seroepidemiology in very low transmission settings in the Peruvian Amazon

**DOI:** 10.1038/s41598-024-52239-5

**Published:** 2024-02-02

**Authors:** Bryan Fernandez-Camacho, Brian Peña-Calero, Martina Guillermo-Roman, Jorge Ruiz-Cabrejos, Jose Luis Barboza, Lucia Bartolini-Arana, Antony Barja-Ingaruca, Hugo Rodriguez-Ferrucci, Veronica E. Soto-Calle, Luca Nelli, Isabel Byrne, Monica Hill, Elin Dumont, Lynn Grignard, Kevin Tetteh, Lindsey Wu, Alejandro Llanos-Cuentas, Chris Drakeley, Gillian Stresman, Gabriel Carrasco-Escobar

**Affiliations:** 1 Health Innovation Laboratory, Institute of Tropical Medicine “Alexander von Humboldt”, Universidad Peruana Cayetano Heredia, Lima, Peru; 2 Universidad Nacional de La Amazonía Peruana, Loreto, Peru; 3 Institute of Tropical Medicine “Alexander von Humboldt”, Universidad Peruana Cayetano Heredia, Lima, Peru; 4 Department of Infection Biology, London School of Hygiene and Tropical Medicine, London, UK; 5 School of Biodiversity, One Health and Veterinary Medicine, University of Glasgow, Glasgow, UK; 6 University of Surrey, Guildford, UK; 7 College of Public Health, Epidemiology Concentration, University of South Florida, Tampa, FL USA; 8 Scripps Institution of Oceanography, University of California San Diego, San Diego, CA USA

**Keywords:** Epidemiology, Malaria

## Abstract

Despite progress towards malaria reduction in Peru, measuring exposure in low transmission areas is crucial for achieving elimination. This study focuses on two very low transmission areas in Loreto (Peruvian Amazon) and aims to determine the relationship between malaria exposure and proximity to health facilities. Individual data was collected from 38 villages in Indiana and Belen, including geo-referenced households and blood samples for microscopy, PCR and serological analysis. A segmented linear regression model identified significant changes in seropositivity trends among different age groups. Local Getis-Ord Gi* statistic revealed clusters of households with high (hotspots) or low (coldspots) seropositivity rates. Findings from 4000 individuals showed a seropositivity level of 2.5% (95%CI: 2.0%-3.0%) for *P. falciparum* and 7.8% (95%CI: 7.0%-8.7%) for *P. vivax*, indicating recent or historical exposure. The segmented regression showed exposure reductions in the 40–50 age group (β1 = 0.043, p = 0.003) for *P. vivax* and the 50–60 age group (β1 = 0.005, p = 0.010) for *P. falciparum*. Long and extreme distance villages from Regional Hospital of Loreto exhibited higher malaria exposure compared to proximate and medium distance villages (p < 0.001). This study showed the seropositivity of malaria in two very low transmission areas and confirmed the spatial pattern of hotspots as villages become more distant.

## Introduction

Between 1955 and 2021, a total of 40 countries were certified by the World Health Organization (WHO) as malaria-free. This status is achieved when endemic countries report zero indigenous cases of malaria for at least three consecutive years^1^. Paraguay, Argentina, and El Salvador obtained this certification in Latin America during that period^2^. The consistent progress in eliminating malaria in this region can be attributed to the implementation of international strategies such as the WHO Global Technical Strategy against Malaria^3,4^.

According to the 2022 World Malaria Report, Peru reduced its estimated malaria cases by 17,000 between 2019 and 2021^5^. The prevalence of malaria cases in the country is concentrated in the department of Loreto, located within the Peruvian Amazon, where 86% of all recorded cases were found in 2021^6^. Over the past two decades, two national malaria control initiatives were established, with Loreto as the main site of action. The Andean Countries Border Malaria Control Project (PAMAFRO in Spanish), implemented between 2006 and 2010, achieved an 80% reduction in cases. However, after the conclusion of PAMAFRO activities, there was an increase in the number of cases^7^. In Loreto specifically, the number of malaria cases surged from 25,084 in 2012 to 54,342 in 2016. Nevertheless, implementing the Zero Malaria Plan (PMC in Spanish) from 2017 to 2021 led to a significant decline in cases, resulting in a reduction of 75% by 2020^8,9^.

In January 2022, the Peruvian Ministry of Health (MINSA in Spanish) approved the plan to eliminate malaria in Peru for 2022–2030. The plan set two important goals: reducing cases by 90% by 2030 and eliminating residual malaria from the country by 2045^10^. The initial phase of this plan focuses on controlling the spread of the disease through reactive case detection in remote areas with low transmission. Factors related to malaria persistence in these areas include *Plasmodium* species (spp) circulating, healthcare-seeking behavior, vector diversity, vector dynamics, sociodemographic determinants, and human mobility patterns^7,11–13^.

In Loreto, there is a high heterogeneity of malaria transmission at the district level, where districts function as the third-level administrative subunits, with areas of both high and low transmission^14^. In regions with high malaria transmission, factors such as climate variables, low levels of treatment adherence, social factors, and limited access to health services contribute to an increased incidence rate of malaria^15^. Similarly, in semi-isolated villages with low malaria transmission, variables such as age, circulating *Plasmodium* spp., and previous positive diagnoses are key factors in malaria transmission^16^. Furthermore, in low transmission settings or areas where elimination efforts have been implemented, human movement plays a crucial role^13,17^.

Serological assays, which detect antibodies to malaria parasites, are considered a robust tool for identifying recent or historical malaria exposure^18^. Antibodies that correlate to recent (within one year) and historical exposure (within 20 years) to *P. falciparum* and *P. vivax* have been characterised^19–21^, providing a sensitive method to assess transmission. Estimating recent exposure within a population can be particularly useful in low transmission areas where traditional methods of diagnosing malaria, such as microscopy or rapid diagnostic tests, may not be sensitive enough to detect low numbers of infections. Serology offers insight into a wider timeframe than traditional diagnostics capturing active infections^22^.

This study aims to evaluate malaria exposure through a panel of serological markers and examine factors such as village proximity to healthcare establishments as an indicator of healthcare accessibility, household spatial distribution, and seropositivity patterns across different age groups in two very low transmission areas of Loreto.

## Methods

### Study design

A cross-sectional analytical study was conducted in two districts, Indiana and Belen, located in the Loreto department. A total of 38 villages within the study area were selected using a three-stage random sampling method as described in Supplementary Methods. From October to December 2021, a full census of inhabitants in the selected households was conducted, and data such as geographic coordinates, structured questionnaires, and blood samples were collected.

### Study area and population

The department of Loreto covers an extensive area of approximately 374000 square kilometers, accounting for over 28% of the total territory of Peru. It is divided into eight provinces and 53 districts, with Iquitos as its capital^23^. According to the 2017 census, the estimated population of Loreto was 883510 inhabitants. In 2021, 33.1% of the population lived in poverty, while 6.9% experienced extreme poverty. Additionally, less than 50% of households had access to basic services such as electricity and water sanitation^24^.

**Figure 1:**
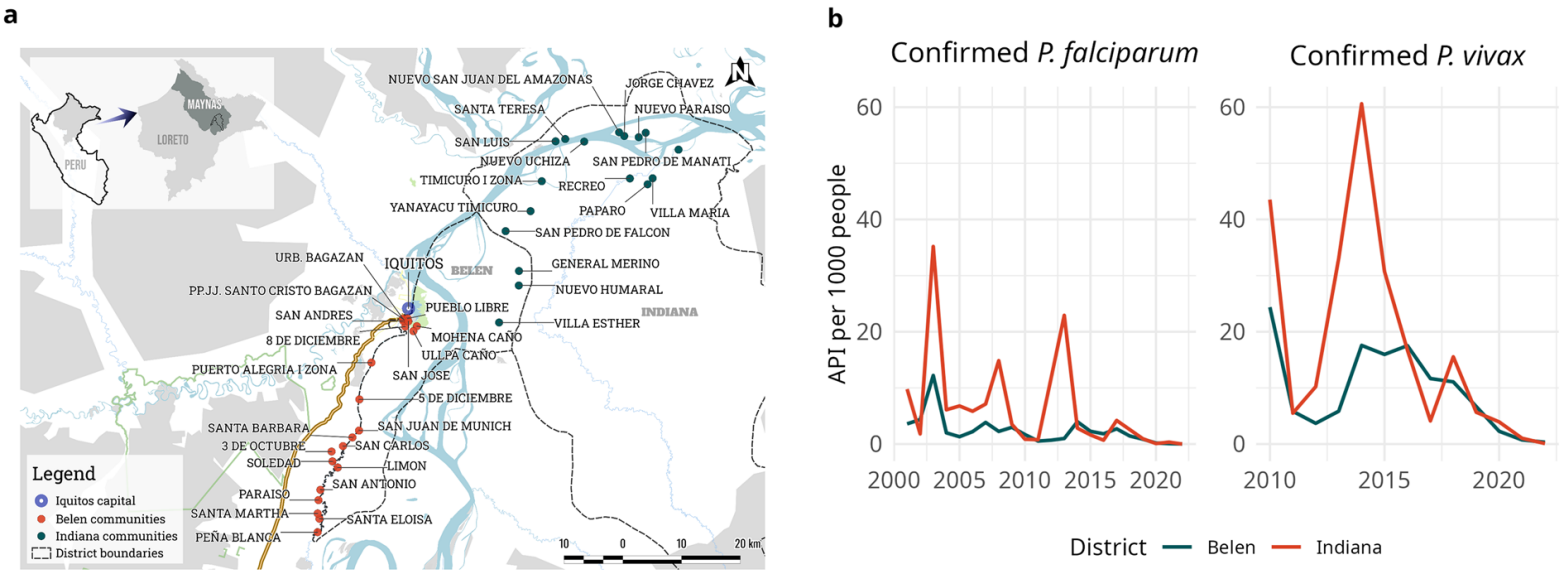
Study area and malaria Annual Parasite Incidence (API) per 1000 people in Indiana and Belen, 2000–2021. The (**a**) map shows 21 villages belonging to Belen district (dots on the left) and 17 villages belonging to Indiana district (dots on the right), the (**b**) API per 1000 people was calculated between 2000 and 2021 in Belen and Indiana for *P. falciparum* and *P. vivax*. The map was generated with QGIS software v3.28.3 (QGIS Development Team, 2016. QGIS Geographic Information System. Open Source Geospatial Foundation Project. http://www.qgis.org/). The line plot was generated with R software v.4.1.2 (R Development Core Team, R Foundation for Statistical Computing, Austria. http://www.R-project.org/).

Very low transmission areas (< 100 cases per 1000 population Annual Parasite Incidence [API])^25^ within the Loreto department were identified as target areas (Supplementary Fig. 1). Following the initial assessment, the districts of Indiana and Belen were selected as the study area (Fig. 1a).

According to the 2017 census, Indiana district has a population of 10134 inhabitants, with 62.3% residing in rural areas. Among the population aged 15 years and older (N = 5963), 44.2% had primary education, while 1.6% had complete university studies^26^. The district experienced peaks of *P. falciparum* API in 2003, 2008 and 2013, as well as peaks of *P. vivax* in 2010 and 2014 **(**Fig. 1b**)**. However, both species showed a decreasing trend of API. The villages in the study area were accessible via the Mazan River, requiring 2–7 h of travel from Iquitos.

In Belen district, the total census population is 64488 inhabitants, with 87.6% in urban areas. Among individuals aged 15 years and older (N = 42727), 23.6% had primary education and 7.7% had complete university studies^26^. Peaks in *P. falciparum* API occurred in 2003, 2007, and 2014 while *P. vivax* peaks were observed in 2010 and 2014, maintaining similar values until 2015 (Fig. 1b). Both species exhibited decreasing trends in API. Villages in this district were accessible via the Canta Gallo road and the Itaya River, with travel times of approximately 1–6 h from Iquitos.

During the pandemic, numerous health activities in Loreto were impacted by the redirection of resources from other diseases to address COVID-19. This resulted in delays in reporting confirmed and tested malaria cases, which affected data availability for decision-making. Furthermore, malaria control efforts, including active detection and vector control, were postponed due to COVID-19^27^.

### Sampling strategy

A stratified three-stage cluster sampling design (Supplementary Fig. 2) was used to select the study subjects. The districts served as strata, and clusters were randomly selected at three levels. In the first stage, the health facilities in each district were classified into four categories of distance (proximate, medium, long, and extreme distance) from the Regional Hospital of Loreto (RHL) and one health facility was randomly selected in each category. Additionally, we defined the health facility catchment area according to their health care report. One health facility in Indiana was excluded as its catchment area had a combined population of less than 400, a prerequisite for inclusion. In the second stage, the health facilities were classified as big or medium-size, according to the population in their catchment area, to identify the number of villages to be sampled in each health facility. In the third stage, households and individuals were censused in those villages with less than 500 inhabitants, while in villages with 500 or more inhabitants, a random selection of households was applied, and all individuals in the selected houses were sampled, following proportional allocation based on the sample size. Furthermore, one village in the health facility “Soledad de Villa Belen” and an entire health facility “Belen de Villa Belen” with four villages in its catchment area were added due to access and availability issues with the initially chosen sample. We included all participants older than three months of age after completion of written informed consent or assent, and excluded individuals younger than three months old because parents may have considered the blood collection procedures inappropriate for this age group. All villages were located within the catchment area of the health facilities of Indiana and Belen.

### Data collection

#### Standardized questionnaires and georeferencing

Household questionnaires were administered to the head of household or immediate caregiver. The questionnaire had one section dedicated to gathering household information and living conditions, such as the total number of household members, availability of insecticide-treated bed nets, household type, main water source, possession of household appliances, and presence of animals.

Individual questionnaires were given to each present household member. The questionnaire consisted of three sections aimed at collecting information on symptoms, medication usage, and healthcare-seeking behavior; factors related to malaria transmission; and human mobility patterns.

All households included in the study were georeferenced using a Garmin 66 s Global Position System (GPS) device.

#### Blood sample collection

Blood samples were collected through finger prick and stored on slides and filter paper. Thin and thick smears were performed for samples on slides, while serology was conducted for samples on filter paper. The analysis of slide samples was performed by the Universidad Peruana Cayetano Heredia fieldwork team, while the London School of Hygiene and Tropical Medicine analyzed the filter paper samples.

### Laboratory procedures

#### Microscopy

Slides were evaluated by expert microscopists during field work. Thick and thin smears were stained with 10% Giemsa for 10 min and examined for parasite density by counting the number of asexual parasites per 200 leukocytes (L) and assuming a concentration of 8000 L/μl. A sample was considered negative if no parasites were detected after examination of 100 microscopy fields^28^.

#### Quantitative real-time PCR (qPCR)

Pieces of filter paper with biological samples were cut into ~ 6 mm^2^ sections for extraction of parasite genomic DNA using the E.Z.N.A.® Blood DNA Kit (Omega Bio-tek®, USA), following the manufacturer's instructions, and stored at 4 °C for immediate use in qPCR and at -20 °C for future use. Diagnostic qPCR was performed following the protocol described by Mangold et al^29^, which consists of a semi-nested multiplex PCR (SnM-PCR) that amplifies the 18S rRNA sequence of *Plasmodium* species. This procedure was repeated for *P. vivax* as *P. falciparum* and quantification of parasitaemia was performed by comparing Ct values with a standard curve ranging from 2 × 10^6^ to 2 × 10^0^ copies. According to current MINSA therapeutic guidelines^30^, parasite carriers detected by qPCR were not treated unless they became positive by microscopy within the next few days or weeks.

#### Serology

Discs of approximately 3 mm in diameter were cut from the dried blood spot samples using a hole puncher (Rayher 8932400) and each was transferred to an individual well in a 96-well plate (Corning™ 3958, USA). One day prior to processing, antibodies were eluted by adding 400 µL of elution buffer (1 × Phosphate-buffered saline (PBS), 0.05% Tween, 0.5% bovine serum albumin (BSA), 0.02% sodium azide, 0.5% polyvinyl alcohol (PVA), 0.5% polyvinyl pyrrolidone (PVP), 0.1% casein, 15.25 ug/mL *E.coli* lysate) to each well (1/400 dilution), and the plates were incubated for 18 h at 4 °C with shaking (Stuart™ microtitre plate shaker; 500 RPM).

A previously optimised panel of four *P. vivax* and seven *P. falciparum* blood-stage antigens associated with historical and recent malaria exposure **(**Supplementary Table 1**)** were coupled to Luminex (Luminex Corporation, Austin, TX, USA) MagPlex® microspheres. Glutathione S-transferase (GST) and tetanus toxoid were included as controls. IgG antibody responses were measured using the Luminex serological assay as described previously^31^. Briefly, antigen-coupled microspheres were co-incubated with 50 µL of eluted samples (1/400 dilution) for 90 min. Two control curves comprising 5-point, fivefold serial dilutions (1/50, 1/250, 1/1250, 1/6250, 1/31,250) of pooled sera from hyper-immune individuals in Kenya (CP3) and Peru (S1) were added to each plate as *P. falciparum* and *P. vivax* positive controls, respectively. The WHO reference reagents for anti-malaria human serum, 10/198 (*P. falciparum*) and 19/198 (*P. vivax*), were also included as positive controls (1/400 dilution). Serum samples from Public Health England (PHE) European blood donors were included as malaria-naïve controls (1/400 dilution). Wells exclusively containing elution buffer served as blanks in each plate for removal of background signal. Following sample incubation, plates were washed with 1xPBS/Tween and incubated with 1/200 AffiniPure Goat Anti-Human IgG R-Phycoerythrin-conjugated secondary antibody (Jackson Immuno Research Laboratories, USA). After a final wash, 100 µL of 1xPBS was added and median fluorescence Intensity (MFI) values were recorded using the MAGPIX© instrument.

### Classification of seropositivity

Individuals were classified as seropositive or seronegative for long-term (historical) and short-term (recent) exposure to *P. falciparum* and *P. vivax* based on their MFI values for the antigens listed in Supplementary Table 1. These antigens were chosen based on their known immunogenicity and longevity in the immune system. Pf/PvMSP119 and Pf/PvAMA1 persist in the blood for up to 20 years post-exposure, serving as a proxy for historical exposure in an individual’s lifetime. On the other hand, Etramp5.Ag1 and PvRBP2b are shorter-lived in the blood and are used to represent recent exposure within the past 6–12 months^18,19,32^.

To classify individuals as seropositive or seronegative for exposure, unsupervised machine learning K-means clustering algorithms were applied to the MFI values of each antigen independently. This method of classifying MFI values has been used in neglected tropical diseases and malaria studies^33,34^. The method partitions data into a predetermined number of clusters, and the optimal number of clusters for each antigen was determined using within-cluster sum of squares and average silhouette testing^35^. For a specific antigen, individuals grouped within the cluster of higher MFI values were classified as seropositive, and individuals within the cluster of lower MFI values were defined as seronegative. If an individual was seropositive to Etramp5Ag1 or PvRBP2b, they were defined as seropositive for recent exposure to *P. falciparum* or *P. vivax*, respectively. Historical exposure to *P. falciparum* was determined by seropositivity to PfMSP119 and/or PfAMA1. Similarly, seropositivity to PvMSP119 and/or PvAMA1 indicated historical exposure to *P. vivax*.

### Data management

We obtained a total sample of 4000 individuals and 980 households through data collection using the REDCap survey manager application on mobile devices. The data underwent rigorous cleaning using R software v4.1.2 (R Development Core Team, R Foundation for Statistical Computing, Austria), which involved processes of data cross-validation, in-range value checks, duplication of information and others (Supplementary Fig. 3).

All villages in the study were classified into four distance categories according to the district to which they belong: proximate distance (Belen range 144–747 m; Indiana range 766–2382 m), medium distance (Belen range 877–2206 m; Indiana range 2755–4467 m), long distance (Belen range 7162–30,716 m; Indiana range 4588–5916 m), and extreme distance (Belen range 31,354–42,830 m; Indiana range 7138–13,101 m). This classification was based on the distances from each village to two key locations: their health facility or the RHL. The shortest calculated distance to either location was recorded for each village. The villages were then ordered from smallest to largest distance, ensuring an equal number of villages in each category.

### Statistical analysis

Statistical analysis was performed with the R software v.4.1.2 and QGIS software v3.28.3 was used to generate the study area map.

Categorical descriptive variables were presented as cross-tabulations with district or malaria positivity, and associations were examined using the Fisher test with p-value calculation through Monte Carlo simulation. Numerical variables were summarized using the median and the Interquartile Range (IQR), and differences between calculated medians were assessed using the Mann–Whitney U test. Descriptive plots were generated to observe changes in malaria seropositivity across village, district, and distance categories.

When evaluating the seropositivity through the age groups graphically, a probable change of trend was observed in some age points, therefore, it was considered to complement it with an analysis technique that allows evaluating more than one regression adjustment at the same time in the data, such as segmented regression^36^. This analysis was applied to determine the age group at which a change in seropositivity trend occurs for each *Plasmodium* spp*.*^37^ Prior to the analysis, the eight age groups were recoded taking values from one to eight, in order to be able to estimate the appropriate breakpoint, and subsequently relocate the age group to which it belongs. The breakpoint was determined iteratively, aiming to maximize the likelihood function and improve the model's fit to the observed data. If the age is below an estimated breakpoint (${U1}_{age}$), the original linear regression equation and its coefficients are used. Conversely, a modified equation is derived for age variables exceeding the estimated breakpoint (${U1}_{age}$).

$${\text{For}}\quad age\le {U1}_{age}:$$

$$Seropositivity= \beta 0+ \beta 1\times age+\varepsilon$$

$${\text{For}}\quad age>{U1}_{age}:$$

$$Seropositivity=\left(\beta 0+ \beta 1\times {U1}_{age}\right)+\left(\beta 1+\beta U1\right)+(age-{U1}_{age})+\varepsilon$$

where $\beta 0$ represents the intercept, $\beta 1$ represents the slope before the breakpoint ($age\le {U1}_{age}$), $\beta U1$ represents the change in slope after the breakpoint ($age>{U1}_{age}$), and $\varepsilon$ represents the random error term.

Additionally, the exposure to malaria in different distance groups was evaluated using the Kruskal Wallis test. Subsequently, multiple comparisons were conducted using the post-hoc Dwass-Steel-Critchlow-Fligner (DSCF) method, which incorporates a single-step correction for p-values.

### Spatial analysis

To identify local patterns and clusters of high- and low-level malaria seropositivity rate in the Indiana and Belen districts, we conducted a local spatial autocorrelation analysis using the Getis-Ord Gi* statistics in the *spdep* package in R^38^. Prior to the analysis, neighboring households were identified on the basis of geographic coordinates and the maximum distance between them in each district was determined. Then, neighbors were recalculated within an interval from zero to the maximum distance and spatial weights were calculated to assess the importance of close neighbors in each reference household. From that previous evaluation, the Getis-Ord Gi* statistic was calculated, estimated in Z values with their respective 90%, 95%, and 99% confidence levels to identify hot and cold spots^39–41^. This identification allows us to evaluate the grouping of observations (households) that share common characteristics with respect to their malaria seroprevalence and the geographic position where they are located. Thus, hot spots are made up of households which are geographically close together with higher seropositivity, and cold spots are made up of households which are close together and have low or zero seropositivity values.

### Ethics approval

The study protocol was registered and approved by the Ethics Committee of Universidad Peruana Cayetano Heredia (approval number 201615) and the London School of Hygiene and Tropical Medicine (approval number 19167) prior to implementation. The study adhered to the ethical principles outlined in the Declaration of Helsinki.

All participants provided written informed consent, and for individuals falling below the age of 18 years but exceeding three months, informed assent and consent from a parent or guardian were obtained. Participants had the right to voluntarily withdraw from the study and have their samples removed at any time. All data collected from primary sources was anonymised and devoid of identifiers. The geographic coordinates were used exclusively for analysis purposes and underwent a dispersion procedure thereafter.

## Results

### Characteristics of study population

A total of 4000 individuals were included in the analysis. The participants, who were predominantly men (51.7%), had a median age of 20 years (IQR: 9.3–45.0). The most frequent education level was primary education (48.2%) and the main economic activities were being a student (28.7%), forest related (26.2%), none (21.7%), and housewives (16.8%). Significant differences (p < 0.001) were observed between the two districts for trip in the last month, frequency of never using mosquito nets, self-reported symptoms of muscle or joint pain and self-reported malaria (Table 1).

**Table 1:** Characteristics of study subjects.

Variable	n*	Belen, n = 2029	Indiana, n = 1971	Overall, n = 4000	p
Sex					
Male	4000	1024 (50.5%)	1045 (53.0%)	2069 (51.7%)	0.107
Female		1005 (49.5%)	926 (47.0%)	1931 (48.3%)	
Age groups					
(0–10)	3988	544 (26.9%)	547 (27.8%)	1091 (27.4%)	0.676
(10–20)		453 (22.4%)	471 (24.0%)	924 (23.2%)	
(20–30)		206 (10.2%)	174 (8.9%)	380 (9.5%)	
(30–40)		227 (11.2%)	208 (10.6%)	435 (10.9%)	
(40–50)		182 (9.0%)	181 (9.2%)	363 (9.1%)	
(50–60)		172 (8.5%)	174 (8.9%)	346 (8.7%)	
(60–70)		117 (5.8%)	103 (5.2%)	220 (5.5%)	
(70 +)		122 (6.0%)	107 (5.4%)	229 (5.7%)	
Education level					
No schooling	3997	382 (18.8%)	383 (19.4%)	765 (19.1%)	0.546
Primary school		971 (47.9%)	957 (48.6%)	1928 (48.2%)	
Secondary school		618 (30.5%)	588 (29.8%)	1206 (30.2%)	
Higher education		56 (2.8%)	42 (2.1%)	98 (2.5%)	
Economic activities					
None	3997	458 (22.6%)	410 (20.8%)	868 (21.7%)	< 0.001
Forest related		456 (22.5%)	591 (30.0%)	1047 (26.2%)	
Trader		87 (4.3%)	17 (0.9%)	104 (2.6%)	
Housewife		379 (18.7%)	294 (14.9%)	673 (16.8%)	
Student		542 (26.7%)	606 (30.8%)	1148 (28.7%)	
Others		105 (5.2%)	52 (2.6%)	157 (3.9%)	
Trip in the last month					
No	3998	1925 (94.9%)	1684 (85.5%)	3609 (90.3%)	< 0.001
Yes		100 (4.9%)	281 (14.3%)	381 (9.5%)	
Don't know/no answer		4 (0.2%)	4 (0.2%)	8 (0.2%)	
Usual places to bathe					
Bathroom inside the dwelling	3988	392 (19.4%)	63 (3.2%)	455 (11.4%)	< 0.001
Bathroom outside the dwelling		278 (13.7%)	99 (5.0%)	377 (9.5%)	
In the countryside/river		1327 (65.6%)	1710 (87.0%)	3037 (76.2%)	
Other		26 (1.3%)	93 (4.7%)	119 (3.0%)	
Place to sleep last night					
Inside the dwelling	3995	2018 (99.6%)	1961 (99.6%)	3979 (99.6%)	> 0.999
Outside the dwelling		8 (0.4%)	7 (0.4%)	15 (0.4%)	
Don't know/don't answer		1 (0.0%)	0 (0.0%)	1 (0.0%)	
Mosquito net	3990	1995 (98.5%)	1960 (99.8%)	3955 (99.1%)	< 0.001
Frequency of Mosquito Net					
Never	3988	83 (4.1%)	18 (0.9%)	101 (2.5%)	< 0.001
Every day		1939 (95.8%)	1944 (99.0%)	3883 (97.4%)	
Sometimes		0 (0.0%)	0 (0.0%)	0 (0.0%)	
Don't know/don't answer		2 (0.1%)	2 (0.1%)	4 (0.1%)	
Self-reported malaria					
No	3995	1352 (66.7%)	1466 (74.5%)	2818 (70.5%)	< 0.001
Yes		652 (32.2%)	498 (25.3%)	1150 (28.8%)	
Don't know/no answer		22 (1.1%)	5 (0.3%)	27 (0.7%)	
Symptoms or signs in the last month					
Fever	3995	218 (10.8%)	159 (8.1%)	377 (9.4%)	0.004
Headache	4000	210 (10.3%)	206 (10.5%)	416 (10.4%)	0.918
Muscle or joint pain	4000	110 (5.4%)	166 (8.4%)	276 (6.9%)	< 0.001
General malaise	4000	110 (5.4%)	162 (8.2%)	272 (6.8%)	< 0.001
*P. falciparum* exposure**					
Negative	4000	1954 (96.3%)	1947 (98.8%)	3901 (97.5%)	< 0.001
Positive		75 (3.7%)	24 (1.2%)	99 (2.5%)	
*P. vivax* exposure**					
Negative	4000	1822 (89.8%)	1865 (94.6%)	3687 (92.2%)	< 0.001
Positive		207 (10.2%)	106 (5.4%)	313 (7.8%)	
Distance category to the regional hospital or health facilities					
Proximate distance	4000	685 (33.8%)	664 (33.7%)	1349 (33.7%)	0.285
Medium distance		366 (18.0%)	326 (16.5%)	692 (17.3%)	
Long distance		500 (24.6%)	531 (26.9%)	1031 (25.8%)	
Extreme distance		478 (23.6%)	450 (22.8%)	928 (23.2%)	

Fisher's Exact Test for count data is applied. Continuous data are presented as median (interquartile range) and Mann–Whitney U test. *Some variables could not sum 4000 individuals due to missing data. **Overall *Plasmodium* species exposure was determined by the presence of a serological marker of recent or historical exposure.

### Laboratory and questionnaire findings

All samples were negative for malaria in microscopy and qPCR. However, we found an overall seropositivity rate of 2.5% for *P. falciparum* and 7.8% for *P. vivax* (Table 1).

Regarding the rate of *P. vivax* seropositivity, recent (4.7%) and historical (5.4%) groups of exposure showed a small difference. Furthermore, significant associations (p < 0.001) were found for variables such as sex, district, age groups, and distance categories. In contrast, traveling (trip in the last month) and mosquito nets used were not associated with the *P. vivax* seropositivity rate (p = 0.875 and p = 0.409, respectively) (Table 2).

**Table 2:** Characteristics of study subjects by *Plasmodium* species and types of exposure.

Variable	*Plasmodium Vivax*						*Plasmodium Falciparum*					
	Recent Exposure*			Historical Exposure*			Recent Exposure*			Historical Exposure*		
	Negative, N = 3814	Positive, N = 186	p	Negative, N = 3783	Positive, N = 217	p	Negative, N = 3981	Positive, N = 19	p	Negative, N = 3918	Positive, N = 82	p
Districts												
Belen	1904 (49.9%)	125 (67.2%)	< 0.001	1890 (50.0%)	139 (64.1%)	< 0.001	2017 (50.7%)	12 (63.2%)	0.359	1964 (50.1%)	65 (79.3%)	< 0.001
Indiana	1910 (50.1%)	61 (32.8%)		1893 (50.0%)	78 (35.9%)		1964 (49.3%)	7 (36.8%)		1954 (49.9%)	17 (20.7%)	
Sex												
Male	1922 (50.4%)	147 (79.0%)	< 0.001	1919 (50.7%)	150 (69.1%)	< 0.001	2058 (51.7%)	11 (57.9%)	0.651	2018 (51.5%)	51 (62.2%)	0.058
Female	1892 (49.6%)	39 (21.0%)		1864 (49.3%)	67 (30.9%)		1923 (48.3%)	8 (42.1%)		1900 (48.5%)	31 (37.8%)	
Age groups												
(0–10)	1090 (28.7%)	1 (0.5%)	< 0.001	1091 (28.9%)	0 (0.0%)	< 0.001	1081 (27.2%)	10 (55.6%)	0.289	1086 (27.8%)	5 (6.1%)	< 0.001
(10–20)	920 (24.2%)	4 (2.2%)		916 (24.3%)	8 (3.7%)		922 (23.2%)	2 (11.1%)		907 (23.2%)	17 (20.7%)	
(20–30)	367 (9.7%)	13 (7.0%)		370 (9.8%)	10 (4.6%)		378 (9.5%)	2 (11.1%)		374 (9.6%)	6 (7.3%)	
(30–40)	408 (10.7%)	27 (14.6%)		397 (10.5%)	38 (17.5%)		434 (10.9%)	1 (5.6%)		425 (10.9%)	10 (12.2%)	
(40–50)	328 (8.6%)	35 (18.9%)		316 (8.4%)	47 (21.7%)		361 (9.1%)	2 (11.1%)		350 (9.0%)	13 (15.9%)	
(50–60)	305 (8.0%)	41 (22.2%)		308 (8.2%)	38 (17.5%)		346 (8.7%)	0 (0.0%)		332 (8.5%)	14 (17.1%)	
(60–70)	197 (5.2%)	23 (12.4%)		187 (5.0%)	33 (15.2%)		220 (5.5%)	0 (0.0%)		212 (5.4%)	8 (9.8%)	
(70 +)	188 (4.9%)	41 (22.2%)		186 (4.9%)	43 (19.8%)		228 (5.7%)	1 (5.6%)		220 (5.6%)	9 (11.0%)	
Economic activities												
None	856 (22.5%)	12 (6.5%)	< 0.001	854 (22.6%)	14 (6.5%)	< 0.001	861 (21.6%)	7 (36.8%)	0.509	862 (22.0%)	6 (7.4%)	< 0.001
Forest related	910 (23.9%)	137 (73.7%)		905 (23.9%)	142 (65.4%)		1042 (26.2%)	5 (26.3%)		1005 (25.7%)	42 (51.9%)	
Trader	101 (2.7%)	3 (1.6%)		101 (2.7%)	3 (1.4%)		103 (2.6%)	1 (5.3%)		101 (2.6%)	3 (3.7%)	
Housewife	648 (17.0%)	25 (13.4%)		632 (16.7%)	41 (18.9%)		671 (16.9%)	2 (10.5%)		659 (16.8%)	14 (17.3%)	
Student	1144 (30.0%)	4 (2.2%)		1140 (30.2%)	8 (3.7%)		1144 (28.8%)	4 (21.1%)		1133 (28.9%)	15 (18.5%)	
Others	152 (4.0%)	5 (2.7%)		148 (3.9%)	9 (4.1%)		157 (3.9%)	0 (0.0%)		156 (4.0%)	1 (1.2%)	
Trip in the last month												
No	3439 (90.2%)	170 (91.4%)	0.875	3423 (90.5%)	186 (85.7%)	0.06	3590 (90.2%)	19 (100.0%)	0.269	3532 (90.2%)	77 (93.9%)	0.463
Yes	365 (9.6%)	16 (8.6%)		350 (9.3%)	31 (14.3%)		381 (9.6%)	0 (0.0%)		376 (9.6%)	5 (6.1%)	
Don't know/no answer	8 (0.2%)	0 (0.0%)		8 (0.2%)	0 (0.0%)		8 (0.2%)	0 (0.0%)		8 (0.2%)	0 (0.0%)	
Usual places to bathe												
Bathroom inside the dwelling	446 (11.7%)	9 (4.8%)	< 0.001	438 (11.6%)	17 (7.8%)	0.017	455 (11.5%)	0 (0.0%)	0.130	447 (11.4%)	8 (9.8%)	0.972
Bathroom outside the dwelling	368 (9.7%)	9 (4.8%)		360 (9.5%)	17 (7.8%)		377 (9.5%)	0 (0.0%)		370 (9.5%)	7 (8.5%)	
In the countryside/river	2872 (75.5%)	165 (88.7%)		2855 (75.7%)	182 (83.9%)		3019 (76.1%)	18 (94.7%)		2972 (76.1%)	65 (79.3%)	
Other	116 (3.1%)	3 (1.6%)		118 (3.1%)	1 (0.5%)		118 (3.0%)	1 (5.3%)		117 (3.0%)	2 (2.4%)	
Mosquito Net	3769 (99.1%)	186 (100.0%)	0.409	3739 (99.1%)	216 (99.5%)	> 0.999	3936 (99.1%)	19 (100.0%)	> 0.999	3875 (99.2%)	80 (97.6%)	0.161
Symptoms or signs in the last month												
Fever	347 (9.1%)	30 (16.1%)	0.003	349 (9.2%)	28 (12.9%)	0.093	375 (9.4%)	2 (10.5%)	0.699	364 (9.3%)	13 (15.9%)	0.054
Headache	380 (10.0%)	36 (19.4%)	< 0.001	380 (10.0%)	36 (16.6%)	0.004	415 (10.4%)	1 (5.3%)	0.713	399 (10.2%)	17 (20.7%)	0.005
Muscle or joint pain	251 (6.6%)	25 (13.4%)	< 0.001	252 (6.7%)	24 (11.1%)	0.018	276 (6.9%)	0 (0.0%)	0.637	267 (6.8%)	9 (11.0%)	0.18
General malaise	247 (6.5%)	25 (13.4%)	< 0.001	249 (6.6%)	23 (10.6%)	0.036	272 (6.8%)	0 (0.0%)	0.636	266 (6.8%)	6 (7.3%)	0.823
Self-reported malaria												
No	2786 (73.1%)	32 (17.2%)	< 0.001	2786 (73.7%)	32 (14.7%)	< 0.001	2805 (70.5%)	13 (68.4%)	0.832	2793 (71.4%)	25 (30.5%)	< 0.001
Yes	1000 (26.3%)	150 (80.6%)		968 (25.6%)	182 (83.9%)		1144 (28.8%)	6 (31.6%)		1094 (28.0%)	56 (68.3%)	
Don't know/no answer	23 (0.6%)	4 (2.2%)		24 (0.6%)	3 (1.4%)		27 (0.7%)	0 (0.0%)		26 (0.7%)	1 (1.2%)	
Distance category to the regional hospital or health facilities												
Proximate distance	1343 (35.2%)	6 (3.2%)	< 0.001	1335 (35.3%)	14 (6.5%)	< 0.001	1345 (33.8%)	4 (21.1%)	0.605	1338 (34.2%)	11 (13.4%)	< 0.001
Medium distance	685 (18.0%)	7 (3.8%)		683 (18.1%)	9 (4.1%)		688 (17.3%)	4 (21.1%)		683 (17.4%)	9 (11.0%)	
Long distance	957 (25.1%)	74 (39.8%)		940 (24.8%)	91 (41.9%)		1026 (25.8%)	5 (26.3%)		1004 (25.6%)	27 (32.9%)	
Extreme distance	829 (21.7%)	99 (53.2%)		825 (21.8%)	103 (47.5%)		922 (23.2%)	6 (31.6%)		893 (22.8%)	35 (42.7%)	

Fisher's Exact Test for count data is applied. Continuous data are presented as median (interquartile range) and Mann–Whitney U test. *Some variables could not sum 4000 individuals due to missing data.

In the case of *P.* *falciparum*, the seropositivity rate was low for recent and historical exposure (0.5% and 2.1%, respectively). In the recent exposure group, none of the variables were associated with malaria seropositivity, while in the historical exposure group, significant associations (p < 0.001) were found for distance categories, economic activity, district, age groups and self-reported malaria (Table 2).

Considering the sociodemographic characteristics subdivided by age group, it can be seen that in the youngest group (0 to 20 years old), the number of positive malaria cases is considerably lower than in the general population. Overall, most of the participants are students or have no reported economic activity (93.2%), with some level of education (66.1%). In contrast to the group of adults (20 years and older), where the majority have the economic activity of forest related (49.9%) and housewives (30.7%) and up to 95.9% associated with some level of education (Supplementary Table 2).

When analyzing the seropositivity rate by types of exposure (recent and historical) for both *Plasmodium* spp*.* with the use of antimalarial drugs and whether the person would go to the health facility in case of malaria infection, only a statistically significant association was observed between drug intake and recent *P. vivax* seropositivity (χ^2^ = 4.50, p = 0.034) as outlined in Supplementary Table 3.

**Figure 2:**
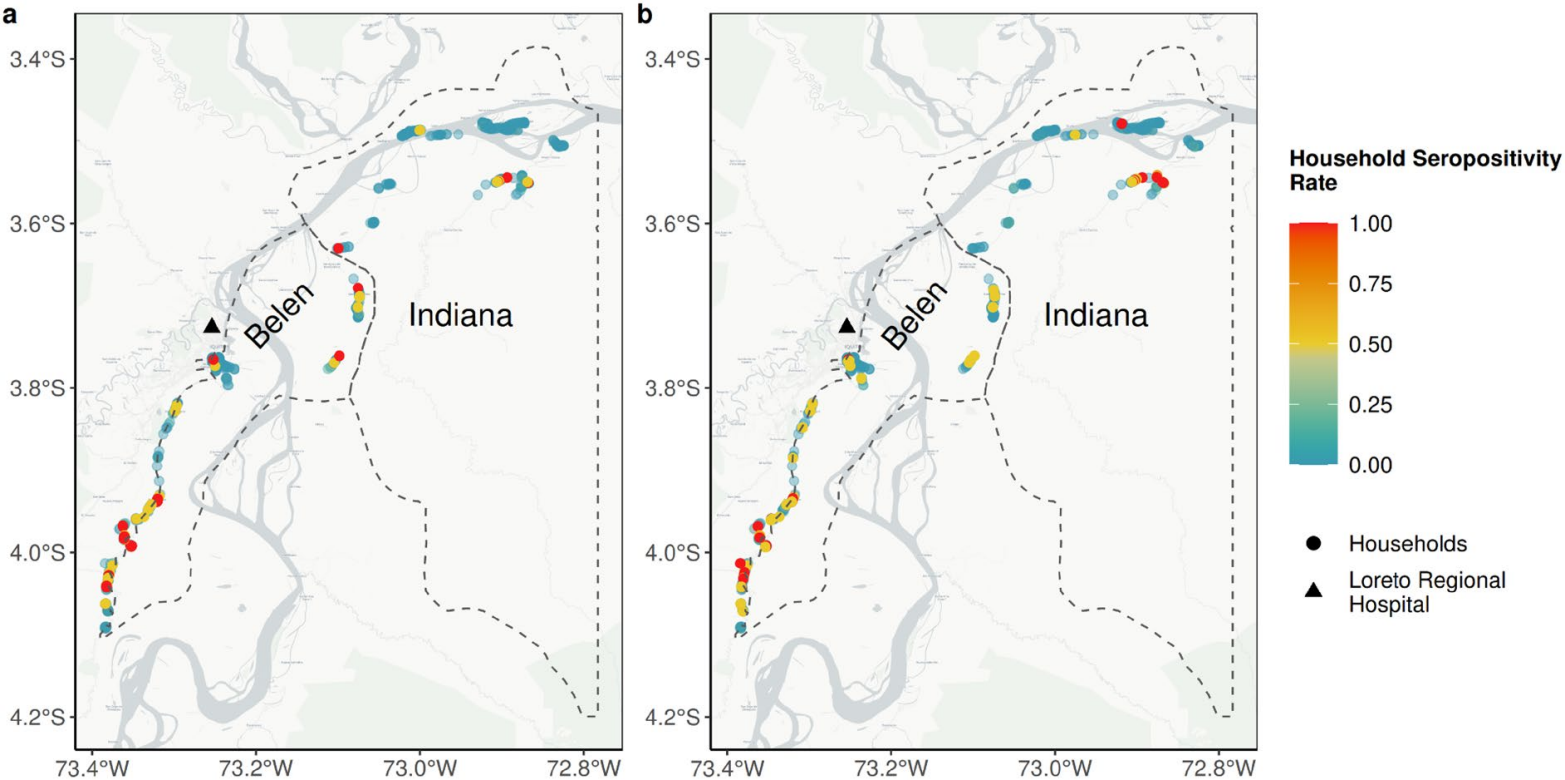
Malaria seropositivity rate by households. Ratio of seropositivity in households for (**a**) recent exposure and (**b**) historical exposure across Belen and Indiana districts represented as dashed lines. Points are households and range in color according to seropositivity ratio, with those in red being households with 100% of their members with historical or recent exposure. The Maps were generated with R software v.4.1.2 (R Development Core Team, R Foundation for Statistical Computing, Austria. http://www.R-project.org/).

**Figure 3:**
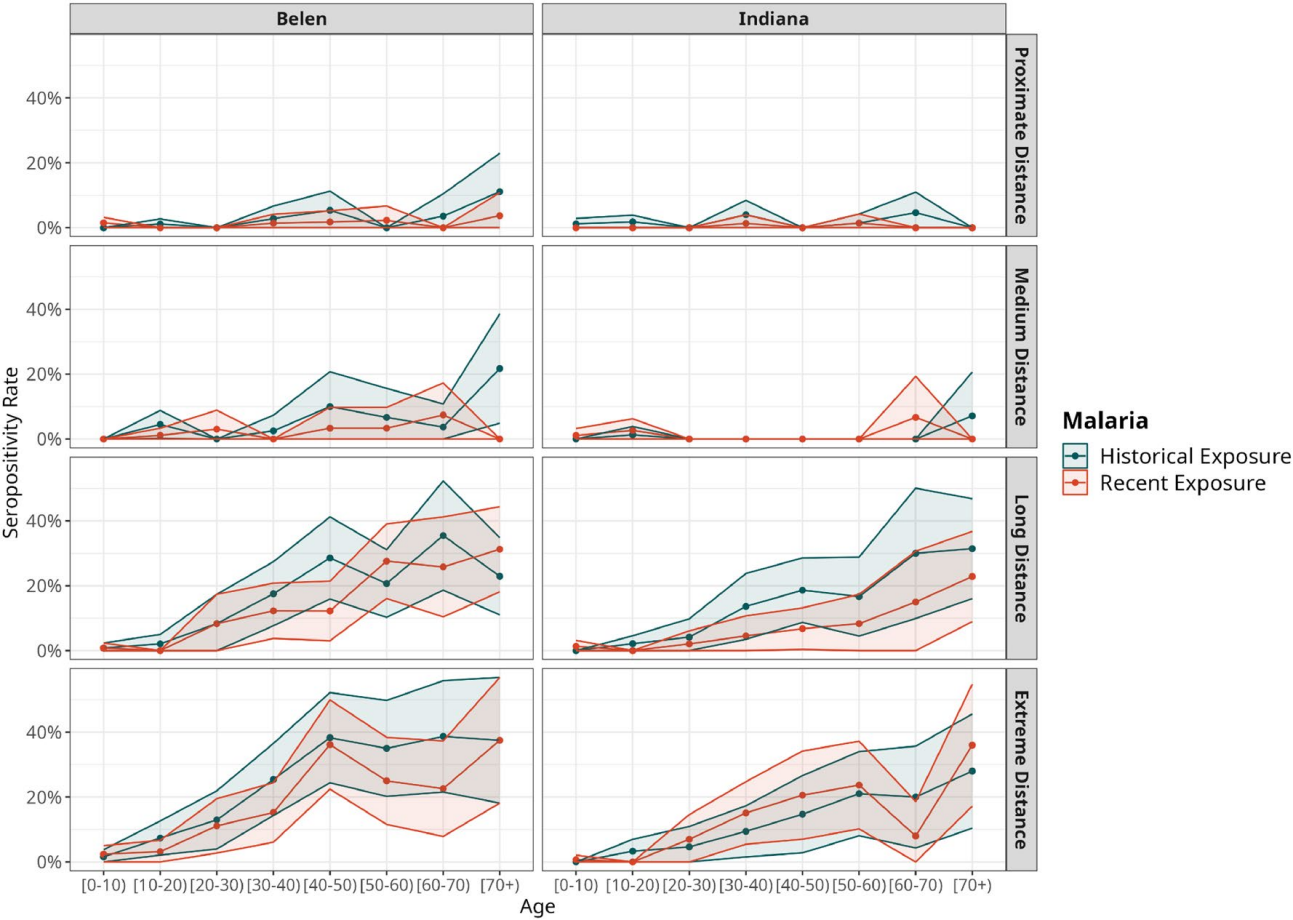
Seropositivity according to time of exposure to malaria by district and distance category of the villages to the Regional Hospital of Loreto (RHL) or their health facilities on a Sampling Basis. Line plot of seropositivity for both types of exposure in the different age groups and categories of distance: proximate, medium, long and extreme distance. The line plots were generated with R software v.4.1.2 (R Development Core Team, R Foundation for Statistical Computing, Austria. http://www.R-project.org/).

Similar patterns of malaria seropositivity were observed in households for both *Plasmodium* spp. in Indiana and Belen. The households located farthest from the RHL exhibited the highest levels of seropositivity rates (Fig. 2). When stratifying by age group and distance category, we found a rise in malaria seropositivity rate as age increased and villages were situated farther away from their health facilities or the RHL. Furthermore, Indiana exhibited lower levels of seropositivity for both types of exposure in the proximate and medium distance categories compared to Belen villages (Fig. 3). We also compared the exposure to any type of malaria (recent or historical) according to the distance level classifications in the villages. Higher exposure was observed in long and extreme distance villages against those classified as proximate (DSCF_long_ = 16.29, p < 0.001; DSCF_extreme distance_ = 18.86, p < 0.001) and medium distance (DSCF_long_ = 10.48, p < 0.001; DSCF_extreme distance_ = 12.53, p < 0.001).

In both *Plasmodium* spp*.*, age groups were found associated with the trend of malaria seropositivity in the segmented linear regression model. In the age group of 40–50 years, there was a significant decline in the seropositivity trend of *P. vivax* (β1 = 0.043, βU1 = − 0.015, p = 0.003). Similarly, the trend of *P. falciparum* showed a downward inflection point in the age group of 50–60 years (β1 = 0.005, βU1 = − 0.003, p = 0.010) (Table 3).

**Table 3:** Segmented linear regression model of malaria seropositivity rate by *Plasmodium* species according to age group. U1 shows the change in slope after breakpoint, 40–50 and 50–60 age groups for *P. vivax* and *P. falciparum*, respectively.

	β	SE	t	p	Break-point	Adjusted R^2^
*P. vivax*						
Intercept	− 0.06	0.023	− 2.628	0.058	40–50	0.946
Age groups	0.043	0.007	6.318	0.003		
U1	− 0.015	0.017	− 0.882			
*P. falciparum*						
Intercept	0.008	0.004	2.328	0.08	50–60	0.899
Age groups	0.005	0.001	4.57	0.01		
U1	− 0.003	0.003	− 1.206			

**Figure 4:**
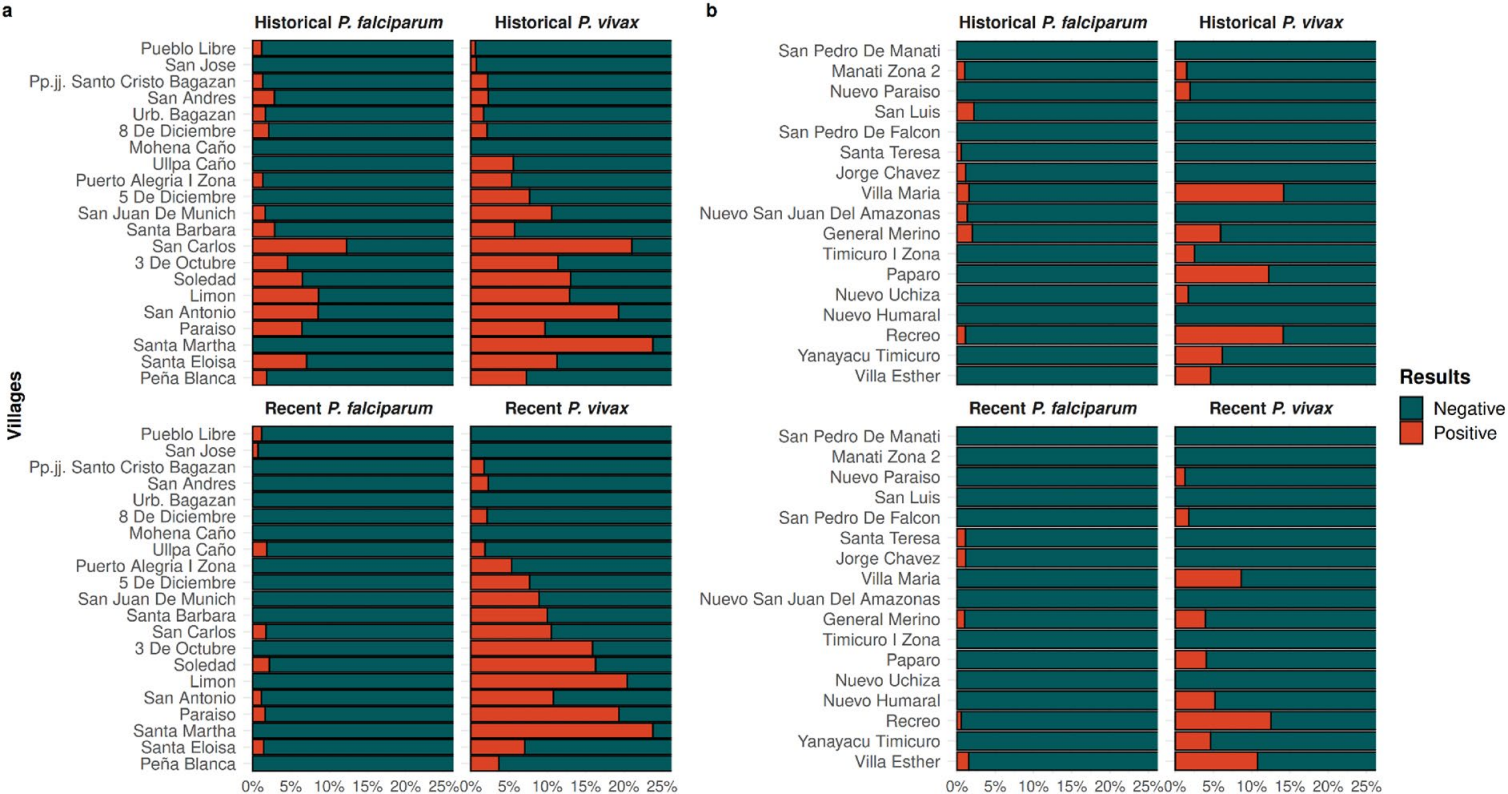
Malaria seropositivity rate by type of exposure and *Plasmodium* species. Bar plot showing malaria serology results according to *Plasmodium* species (*P. falciparum* or *P. vivax*) and time of exposure (recent or historical) in each village for (**a**) Belen and (**b**) Indiana. Villages are ordered according to the distance to their health facility or the Regional Hospital of Loreto (RHL). The bar plots were generated with R software v.4.1.2 (R Development Core Team, R Foundation for Statistical Computing, Austria. http://www.R-project.org/).

In Belen, Santa Martha is the village with more individuals exposed to *P. vivax* in both recent and historical exposure groups. On the other hand, no exposure has been registered in Mohena Caño individuals for both *Plasmodium* spp*.* (Fig. 4a). While in Indiana, high levels of seropositivity are observed in Recreo in both recent and historical exposure to *P. vivax*. Otherwise, San Pedro de Manati was the village with non exposed individuals to both, *P. vivax* and *P. falciparum* (Fig. 4b).

### Spatial analysis

**Figure 5:**
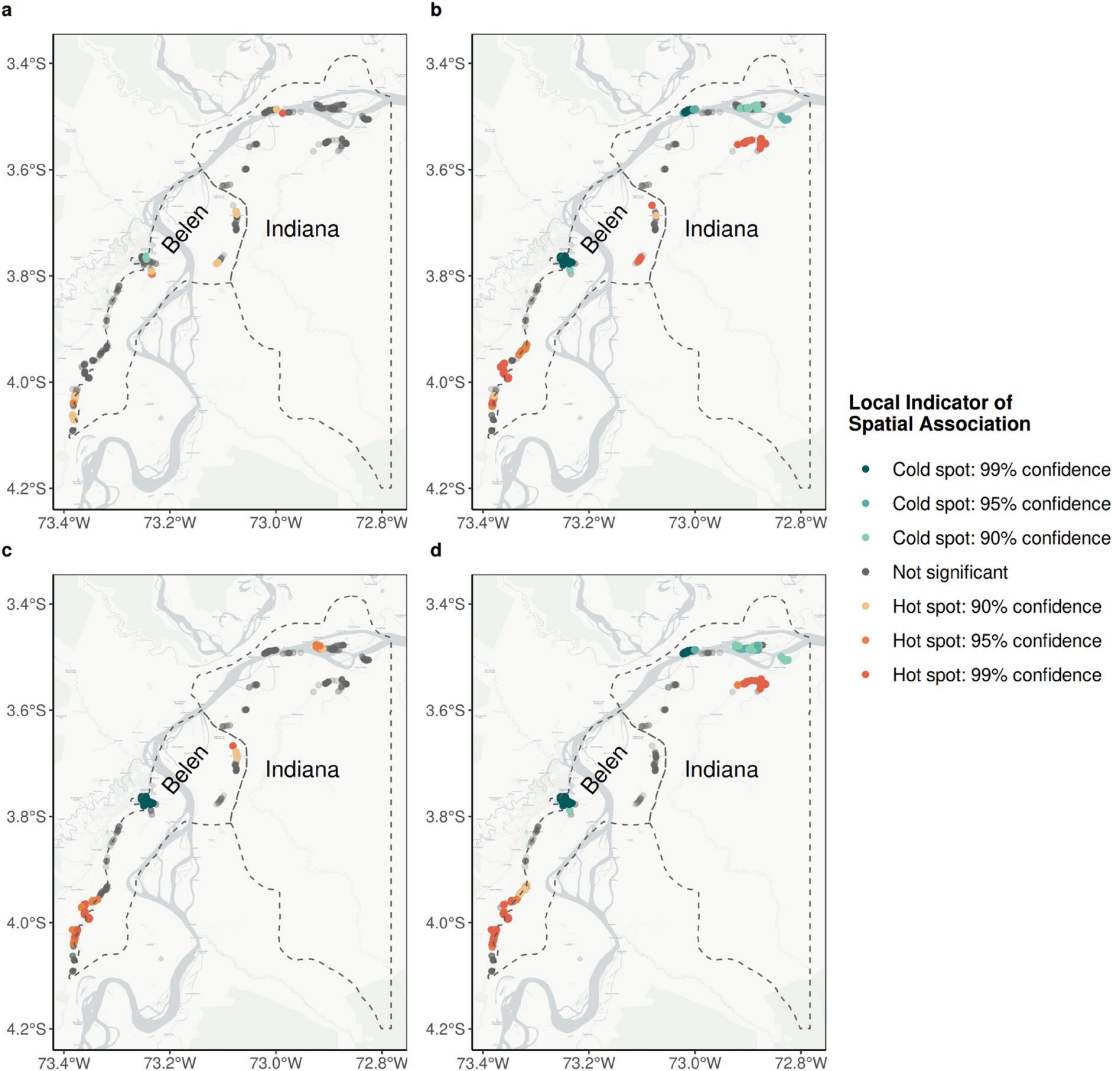
Spatial analysis by type of exposure and *Plasmodium* species. Map showing the locations of households based on the results of the spatial analysis that identifies hot spots and cold spots (with different confidence intervals) for (**a**) Recent *P. falciparum*, (**b**) Recent *P. vivax*, (**c**) Historical *P. falciparum* and (**d**) Historical *P. vivax*. The Maps were generated with R software v.4.1.2 (R Development Core Team, R Foundation for Statistical Computing, Austria. http://www.R-project.org/).

Spatial autocorrelation analysis was run on the seropositivity rate at recent *P. falciparum* exposure according to the district. In both districts, Belen and Indiana, 90.1% and 97.4% of households did not show significant patterns of high or low malaria seropositivity rate clustered spatially (Fig. 5a and Supplementary Fig. 4).

In contrast, recent *P. vivax* had a completely different result. In Belen, 47.2% of the households were identified as cold spots with a 99% confidence level. Particularly, all households, in 6 of 21 villages, were categorized as cold spots located in the center of the district (Fig. 5b and Supplementary Fig. 4). Additionally, hotspots are identified in 30.1% of the remaining households, most of which are found on the district's edge and are farther from the RHL. In Indiana, the proportions of hot and cold spots are lower. About 20% of the households are categorized as hot spots and are located mainly behind the Amazon River, while about 37% of the remaining households are associated with low or absence of *P. vivax* exposure, being considered as cold spots (Fig. 5b and Supplementary Fig. 4).

For historical exposure, similar results have been found. In Belen and Indiana, 47.6% and 41.9% of the households were identified as hot spots, respectively. For *P. vivax,* 26.5% of households were classified as hot spots and 45.0% as cold spots. *P. falciparum* had similar outcomes, with 18.6% of households being categorized as cold spots and 24.8% as hot spots (Fig. 5c,d).

## Discussion

This study makes a valuable contribution to the limited literature on malaria epidemiology in low-transmission settings in the Amazon Region. By analyzing serological markers of *P. vivax* and *P. falciparum*, the study confirmed minimal malaria transmission in the Belen and Indiana districts. However, in long and extreme distance villages, higher seropositivity rate patterns were observed. Furthermore, a pattern of higher seropositivity with increasing age was found for both recent and historical exposure. Additionally, a significant association was identified between seropositivity for *P. vivax* and behavioral malaria risk factors, such as engaging in forest activities and bathing in rivers or countryside. Spatial analysis supported these findings, demonstrating that the hot spots were situated in the most remote regions of both districts.

In other Loreto areas, research on *Plasmodium* spp*.* seropositivity using serological exposure markers (SEM) has been done. In a cross-sectional study carried out in 2018, high seropositivity rate for *P. vivax* in Iquitos and Mazan was found (38.2% and 56.5%, respectively)^42^. However, SEM use is suggested for regions with low malaria transmission, since it is probably less efficient in regions with high transmission^43^. Despite yielding zero positive cases through conventional microscopy and qPCR, we were able to ascertain levels of seropositivity. This enabled us to investigate transmission, underscoring the utility of SEM as a useful tool for malaria surveillance in regions characterized by very low transmission rates, particularly in settings where traditional methods such as microscopy, rapid diagnostic tests, or qPCR may lack the sensitivity required to detect low-level infections or asymptomatic cases^22,44^.

In this study, the villages located in long and extreme distance from their health facilities or the RHL reported higher levels of malaria seropositivity rate than those located in the proximate distance category. Timely access to health facilities for malaria diagnosis and treatment remains an obstacle for people living in remote areas. In 2016, both northern and southern Amazonia were described as having limited accessibility to health services^7^. Exclusively river-based transportation, boat availability, lengthy trip periods, and transportation-related expenditures were some of the obstacles to reaching the most remote study villages. Failure to cover these factors could lead to fragile sustainability of health programs during the malaria elimination phase^4^.

Hot spots of malaria cases are areas where the prevalence of malaria is particularly high^45^. The spatial analysis conducted in this study supported the seropositivity rate pattern observed for both *Plasmodium* species in the Belen and Indiana districts. When stratified by exposure, it was observed that, in both recent and historical exposure, hot spots were located in the most remote areas of the districts. These areas are prone to factors that promote the breeding of *Anopheles* spp*.* mosquitoes, and conditions that increase the risk of infection^46^. Specifically, malaria hotspots in the study villages may arise due to local variables such as poverty, limited healthcare access, inadequate health infrastructure, travel to high-risk regions, and other preventive measures.

Our study revealed distinct inflection points in age-related seropositivity for *P. vivax* and *P. falciparum* at ages 40–50 and 50–60, respectively. In the context of recurrent exposure to the malaria parasite among older individuals, acquired immunity may play a crucial role in diminishing seroprevalence, potentially leading to a reduction in symptom severity and a decrease in seropositivity for malaria-specific antibodies^47^. Moreover, behavioral and environmental changes in older age groups, such as increased indoor activity and mosquito bed net usage, contributed to reduced exposure to malaria vectors, leading to a decline in seroprevalence. The fluctuations observed within the 40–50 age group can be attributed to a complex interplay of factors, including acquired immunity, changes in exposure, waning immunity, age-related variations in immune responses, and demographic and environmental factors. Given the descriptive purpose of the study, a multivariate analysis is needed to uncover the precise causes of age trends.

The COVID-19 pandemic caused disruptions in the delivery of health services, including malaria prevention and treatment interventions. In Peru, there have been delays in the distribution of insecticide-treated bed nets and interruptions in the supply chain of antimalarial drugs, leading to an increase in malaria-related morbidity and mortality^27^. In the year of study (2021), reported cases were lower than in 2019. This may have been possible due to movement restrictions during the pandemic^48^. However, these restrictions may also have affected access to care, reducing cases reported from health facilities. The study showed that historical exposure frequencies were slightly different from recent exposure, indicating that cases remained stable despite the pandemic in very low transmission areas.

The limitations of this study are related to recall bias, due to the fact that some variables are self-reported. To overcome this bias, the time window of the questions was reduced to one month. Additionally, *P. vivax* antigens associated specifically with markers of relapse infection had not yet been identified, limiting the ability to differentiate recent *P. vivax* exposure as novel or relapse infection. Furthermore, not accounting for malaria recrudescent cases in the study may underestimate the ongoing transmission risk, as these cases can contribute to the persistence of the disease in the population. Finally, results from spatial analysis of historical exposure are less definitive. As we define historical exposure as any exposure within the past 20 years, mapping becomes difficult as we cannot tease apart whether exposed individuals are likely to have been exposed within the village they now live, or somewhere else.

In conclusion, the seropositivity rate of *P. vivax* and *P. falciparum* found in Belen and Indiana was consistent with what was expected for areas of very low transmission. In addition, the higher concentration of malaria exposure found in the most remote villages in each district can have important consequences for elimination efforts. These include challenges in accessing and treating affected individuals due to limited resources and infrastructure, and impeding distribution of mosquito nets, indoor residual spraying, and antimalarial medication. Additionally, there is a risk of reintroduction of the disease from neighboring areas with higher transmission rates, even after successful elimination in the region. Thus, ensuring the establishment of effective malaria surveillance and the implementation of malaria control strategies in remote villages can play a crucial role in advancing the efforts towards malaria elimination.

### Supplementary Information

Supplementary Information.

## Data Availability

The datasets used in this study to create the maps and perform the spatial analysis will not be available due to ethics considerations. The seropositivity dataset will be available at https://github.com/healthinnovation/ffi_malaria_seroepidemiology.

## Code availability

Codes to generate tables and figures can be accessed at https://github.com/healthinnovation/ffi_malaria_seroepidemiology.
