# Supplementary material for: Malaria seroepidemiology in very low transmission settings in the Peruvian Amazon
Source: Sci Rep. 2024 Feb 2;14:2806. doi: 10.1038/s41598-024-52239-5 (PMC10837415; doi:10.1038/s41598-024-52239-5)

**Malaria seroepidemiology in very low transmission settings in the Peruvian Amazon**

Bryan Fernandez-Camacho^1,*^, Brian Peña-Calero^1^, Martina Guillermo-Roman^1^, Jorge Ruiz-Cabrejos^1^, Jose Luis Barboza^1^, Lucia Bartolini-Arana^1^, Antony Barja-Ingaruca^1^, Hugo Rodriguez-Ferrucci^2^, Veronica E. Soto-Calle^3^, Luca Nelli^4,5^, Isabel Byrne^4^, Monica Hill^6^, Elin Dumont^4^, Lynn Grignard^4^, Kevin Tetteh^4^, Lindsey Wu^4^, Alejandro Llanos-Cuentas^3^, Chris Drakeley^4^, Gillian Stresman^4,7^, Gabriel Carrasco-Escobar^1,8^

^1^ Health Innovation Laboratory, Institute of Tropical Medicine “Alexander von Humboldt”, Universidad Peruana Cayetano Heredia, Lima, Peru

^2^ Universidad Nacional de la Amazonía Peruana, Loreto, Peru

^3^ Institute of Tropical Medicine “Alexander von Humboldt”, Universidad Peruana Cayetano Heredia, Lima, Peru

^4^ Department of Infection Biology, London School of Hygiene and Tropical Medicine, London, United Kingdom

^5^ School of Biodiversity, One Health and Veterinary Medicine. University of Glasgow, United Kingdom

^6^ University of Surrey, United Kingdom

^7^ College of Public Health, Epidemiology Concentration, University of South Florida, FL, United States of America

^8^ Scripps Institution of Oceanography, University of California San Diego, CA, United States of America

**^*^ Corresponding author:** Bryan Fernandez Camacho: [bryan.fernandez.c@upch.pe](mailto:bryan.fernandez.c@upch.pe)

**SUPPLEMENTARY INFORMATION**

1. **Supplementary Methods**
   1. **Sample Selection**

**Stage I: Health Facilities Selection**

The maximum number of individuals per village was defined at 215; this number can also be understood as a "truncated number". Some inclusion parameters were defined, such as considering eligible villages as those with more than 60 inhabitants. We defined the inclusion of villages in the health facility catchment area using the population health care report. Also, we consider eligible those health facilities whose sum of the truncated number of their affiliated villages is greater than or equal to 400. In addition, the distances between these health facilities and the Regional Hospital of Loreto, which was defined as the starting point, were calculated and health facilities were classified according to quartiles of distance (moderate, close, distant, extra distant) for each district and one health facility per distance quartile was randomly selected for each of the two districts studied.

**Stage II: villages Selection**

In the next stage, the health facilities were classified according to the truncated number; if this value was greater than or equal to 600, the health facility was classified as big size and the objective was to randomize a total of 4 villages. Otherwise, it was classified as regular size and the aim was to randomize up to a total of 5 villages.

**Stage III: Households/Participants Selection**

Finally, in those villages, previously selected, with a population greater than or equal to 500 inhabitants, a proportional random sampling was carried out in order to obtain the households and participants to be surveyed. If the villages had a population of less than 500 inhabitants, a village census was conducted.

All participants underwent a process of malaria symptoms recording and capillary blood sampling (thick smear) for diagnosis of malaria or other pathogens, regardless of the presence of symptoms. Diagnosis was defined by microscopy and serological analysis.

All participants older than 3 months of age were included in the study, after completion of written informed consent or assent. All participants younger than 3 months of age were excluded from the study because parents may have considered the blood collection procedures inappropriate for this age group.

1. **Supplementary Tables**

**Supplementary Table 1.** Malaria antigens used to define *P. falciparum* and *P. vivax* exposure by serology. *IE* infected erythrocyte, *PVM* parasitophorous vacuole membrane.

| **Species** | **Plasmodb**  **Gene ID** | **Antigen** | **Description** | **Location** | **Marker type** |
| --- | --- | --- | --- | --- | --- |
| *P. falciparum* | PF3D7_1133400 | PfAMA1 | *P. falciparum* apical membrane antigen 1 | Merozoite surface | Historical exposure |
| *P. falciparum* | PF3D7_0930300 | PfMSP1_19_ | 19kDa fragment of *P. falciparum* merozoite surface protein 1 | Merozoite surface | Historical exposure |
| *P. falciparum* | PF3D7_1035300 | GLURP R2 | Glutamate-rich protein | All parasite stages | Historical exposure |
| *P. falciparum* | PF3D7_0532100 | Etramp 5 Ag1 | Early transcribed membrane protein 5 antigen 1 | IE / PVM | Recent exposure |
| *P. falciparum* | PF3D7_0402400 | GEXP18 | Gametocyte export protein 18 | Gametocyte | Recent exposure |
| *P. falciparum* | PF3D7_0501100.1 | HSP40 Ag1 | Heat shock protein 40, type II | IE /  Gametocyte | Recent exposure |
| *P. falciparum* | PF3D7_1335400 | RH2.2030 | Reticulocyte binding protein 2 homologue a | Merozoite rhoptry | Protection |
| *P. vivax* | PVX_092275 | PvAMA1 | *P. vivax* apical membrane antigen 1 | Merozoite surface | Historical exposure |
| *P. vivax* | PVX_099980 | PvMSP1_19_ | 19kDa fragment of *P. vivax* merozoite surface protein 1 | Merozoite surface | Historical exposure |
| *P. vivax* | PVX_110810 | PvDBP-RII | *P. vivax* Duffy Binding Protein Region II | Merozoite micronemes |  |
| *P. vivax* | PVX_094255 | PvRBP2b | *P. vivax* Reticulocyte Binding Protein 2b fragment | Merozoite micronemes | Recent exposure |
| *Clostridium tetani* | *--* | Tetanus toxoid | Tetanus toxoid vaccine protein (NIBSC 02/232) | -- | N/A; Internal assay quality control |
| *Schistosoma japonicum* | GST26_SCHJA | GST | Gluthanoid-S-transferase | -- | N/A; GST-tagged protein control |

**Supplementary Table 2.** Characteristics of study subjects by *Plasmodium* species and types of exposure.

| **Variable** | | ***Plasmodium Vivax*** | | | | | | ***Plasmodium Falciparum*** | | | | | | **Overall** |
| --- | --- | --- | --- | --- | --- | --- | --- | --- | --- | --- | --- | --- | --- | --- |
|  |  | **Recent Exposure*** | | | **Historical Exposure*** | | | **Recent Exposure*** | | | **Historical Exposure*** | | |  |
|  |  | **Negative** | **Positive** | **p** | **Negative** | **Positive** | **p** | **Negative** | **Positive** | **p** | **Negative** | **Positive** | **p** |  |
| **Age group: [0-20) years old** | | | | | | | | | | | | | |  |
| **N** |  | 2,010 | 5 |  | 2,007 | 8 |  | 2,003 | 12 |  | 1,993 | 22 |  | 2015 |
| **Districts** | Belen | 992 (49.4%) | 5 (100.0%) | 0.030 | 993 (49.5%) | 4 (50.0%) | > 0.999 | 991 (49.5%) | 6 (50.0%) | > 0.999 | 983 (49.3%) | 14 (63.6%) | 0.203 | 997 (49.5%) |
|  | Indiana | 1,018 (50.6%) | 0 (0.0%) |  | 1,014 (50.5%) | 4 (50.0%) |  | 1,012 (50.5%) | 6 (50.0%) |  | 1,010 (50.7%) | 8 (36.4%) |  | 1018 (50.5%) |
| **Sex** | Male | 1,060 (52.7%) | 4 (80.0%) | 0.378 | 1,059 (52.8%) | 5 (62.5%) | 0.729 | 1,056 (52.7%) | 8 (66.7%) | 0.395 | 1,050 (52.7%) | 14 (63.6%) | 0.39 | 1064 (52.8%) |
|  | Female | 950 (47.3%) | 1 (20.0%) |  | 948 (47.2%) | 3 (37.5%) |  | 947 (47.3%) | 4 (33.3%) |  | 943 (47.3%) | 8 (36.4%) |  | 951 (47.2%) |
| **Education Level** | No schooling | 682 (34.0%) | 0 (0.0%) | 0.076 | 682 (34.0%) | 0 (0.0%) | 0.136 | 677 (33.9%) | 5 (41.7%) | 0.212 | 680 (34.2%) | 2 (9.1%) | 0.045 | 682 (33.9%) |
|  | Primary school | 852 (42.5%) | 5 (100.0%) |  | 852 (42.5%) | 5 (62.5%) |  | 850 (42.5%) | 7 (58.3%) |  | 845 (42.5%) | 12 (54.5%) |  | 857 (42.6%) |
|  | Secondary school | 461 (23.0%) | 0 (0.0%) |  | 458 (22.9%) | 3 (37.5%) |  | 461 (23.1%) | 0 (0.0%) |  | 453 (22.8%) | 8 (36.4%) |  | 461 (22.9%) |
|  | Higher education | 12 (0.6%) | 0 (0.0%) |  | 12 (0.6%) | 0 (0.0%) |  | 12 (0.6%) | 0 (0.0%) |  | 12 (0.6%) | 0 (0.0%) |  | 12 (0.6%) |
| **Economic Activities** | None | 737 (36.7%) | 0 (0.0%) | 0.129 | 737 (36.8%) | 0 (0.0%) | 0.142 | 730 (36.5%) | 7 (58.3%) | 0.235 | 735 (36.9%) | 2 (9.5%) | 0.003 | 737 (36.6%) |
|  | Forest Related | 58 (2.9%) | 0 (0.0%) |  | 58 (2.9%) | 0 (0.0%) |  | 57 (2.9%) | 1 (8.3%) |  | 54 (2.7%) | 4 (19.0%) |  | 58 (2.9%) |
|  | Trader | 5 (0.2%) | 0 (0.0%) |  | 5 (0.2%) | 0 (0.0%) |  | 5 (0.3%) | 0 (0.0%) |  | 5 (0.3%) | 0 (0.0%) |  | 5 (0.2%) |
|  | Housewife | 64 (3.2%) | 1 (20.0%) |  | 65 (3.2%) | 0 (0.0%) |  | 65 (3.3%) | 0 (0.0%) |  | 65 (3.3%) | 0 (0.0%) |  | 65 (3.2%) |
|  | Student | 1,135 (56.6%) | 4 (80.0%) |  | 1,131 (56.4%) | 8 (100.0%) |  | 1,135 (56.8%) | 4 (33.3%) |  | 1,124 (56.5%) | 15 (71.4%) |  | 1139 (56.6%) |
|  | Others | 8 (0.4%) | 0 (0.0%) |  | 8 (0.4%) | 0 (0.0%) |  | 8 (0.4%) | 0 (0.0%) |  | 8 (0.4%) | 0 (0.0%) |  | 8 (0.4%) |
| **Age group: [20+) years old** | | | | | | | | | | | | | |  |
| **N** |  | 1,793 | 180 |  | 1,764 | 209 |  | 1,967 | 6 |  | 1,913 | 60 |  | 1973 |
| **Districts** | Belen | 907 (50.6%) | 119 (66.1%) | < 0.001 | 891 (50.5%) | 135 (64.6%) | < 0.001 | 1,021 (51.9%) | 5 (83.3%) | 0.220 | 975 (51.0%) | 51 (85.0%) | < 0.001 | 1026 (52.0%) |
|  | Indiana | 886 (49.4%) | 61 (33.9%) |  | 873 (49.5%) | 74 (35.4%) |  | 946 (48.1%) | 1 (16.7%) |  | 938 (49.0%) | 9 (15.0%) |  | 947 (48.0%) |
| **Sex** | Male | 856 (47.7%) | 142 (78.9%) | < 0.001 | 853 (48.4%) | 145 (69.4%) | < 0.001 | 995 (50.6%) | 3 (50.0%) | > 0.999 | 961 (50.2%) | 37 (61.7%) | 0.089 | 998 (50.6%) |
|  | Female | 937 (52.3%) | 38 (21.1%) |  | 911 (51.6%) | 64 (30.6%) |  | 972 (49.4%) | 3 (50.0%) |  | 952 (49.8%) | 23 (38.3%) |  | 975 (49.4%) |
| **Education Level** | No schooling | 69 (3.8%) | 13 (7.2%) | < 0.001 | 67 (3.8%) | 15 (7.2%) | < 0.001 | 81 (4.1%) | 1 (16.7%) | 0.279 | 80 (4.2%) | 2 (3.3%) | 0.340 | 82 (4.2%) |
|  | Primary school | 949 (52.9%) | 118 (65.6%) |  | 931 (52.8%) | 136 (65.1%) |  | 1,063 (54.0%) | 4 (66.7%) |  | 1,030 (53.8%) | 37 (61.7%) |  | 1067 (54.1%) |
|  | Secondary school | 690 (38.5%) | 48 (26.7%) |  | 680 (38.5%) | 58 (27.8%) |  | 737 (37.5%) | 1 (16.7%) |  | 717 (37.5%) | 21 (35.0%) |  | 738 (37.4%) |
|  | Higher education | 85 (4.7%) | 1 (0.6%) |  | 86 (4.9%) | 0 (0.0%) |  | 86 (4.4%) | 0 (0.0%) |  | 86 (4.5%) | 0 (0.0%) |  | 86 (4.4%) |
| **Economic Activities** | None | 118 (6.6%) | 12 (6.7%) | < 0.001 | 116 (6.6%) | 14 (6.7%) | < 0.001 | 130 (6.6%) | 0 (0.0%) | > 0.999 | 126 (6.6%) | 4 (6.7%) | 0.243 | 130 (6.6%) |
|  | Forest Related | 849 (47.4%) | 136 (75.6%) |  | 843 (47.8%) | 142 (67.9%) |  | 981 (49.9%) | 4 (66.7%) |  | 947 (49.5%) | 38 (63.3%) |  | 985 (49.9%) |
|  | Trader | 95 (5.3%) | 3 (1.7%) |  | 95 (5.4%) | 3 (1.4%) |  | 98 (5.0%) | 0 (0.0%) |  | 95 (5.0%) | 3 (5.0%) |  | 98 (5.0%) |
|  | Housewife | 581 (32.4%) | 24 (13.3%) |  | 564 (32.0%) | 41 (19.6%) |  | 603 (30.7%) | 2 (33.3%) |  | 591 (30.9%) | 14 (23.3%) |  | 605 (30.7%) |
|  | Student | 6 (0.3%) | 0 (0.0%) |  | 6 (0.3%) | 0 (0.0%) |  | 6 (0.3%) | 0 (0.0%) |  | 6 (0.3%) | 0 (0.0%) |  | 6 (0.3%) |
|  | Others | 144 (8.0%) | 5 (2.8%) |  | 140 (7.9%) | 9 (4.3%) |  | 149 (7.6%) | 0 (0.0%) |  | 148 (7.7%) | 1 (1.7%) |  | 149 (7.6%) |

Fisher's Exact Test for Count Data is applied. Continuous data are presented as median (interquartile range) and Mann-Whitney U test. *Some variables could not sum 4,000 individuals due to missing data.

**Supplementary Table 3.** Association of malaria antigen seropositivity with the use of medication and malaria clinic attendance.

| *Plasmodium* Species | Type of Exposure | Antimalarial drugs use | | Going to the health facility in case of malaria | |
| --- | --- | --- | --- | --- | --- |
|  |  | χ²_(1)_ | p | χ²_(1)_ | p |
| *P. falciparum* | Historic | 0.029 | 0.865 | <0.001 | >0.999 |
|  | Recent | 0.031 | 0.860 | <0.001 | >0.999 |
| *P. vivax* | Historic | 0.662 | 0.416 | 1.340 | 0.247 |
|  | Recent | 4.500 | 0.034 | 0.805 | 0.370 |

1. **Supplementary Figures**

**Supplementary Figure 1.** Malaria Annual Parasite Index per 1,000 people in Loreto by district, 2018. Map of Loreto with delimitations by districts according to the Annual Parasite Incidence (API) level of malaria during 2018. A more intense color represents a higher API level, while a lighter shade indicates a lower presence of API. The map was generated with R software v.4.1.2 (R Development Core Team, R Foundation for Statistical Computing, Austria. [http://www.R-project.org/](http://www.r-project.org/)).


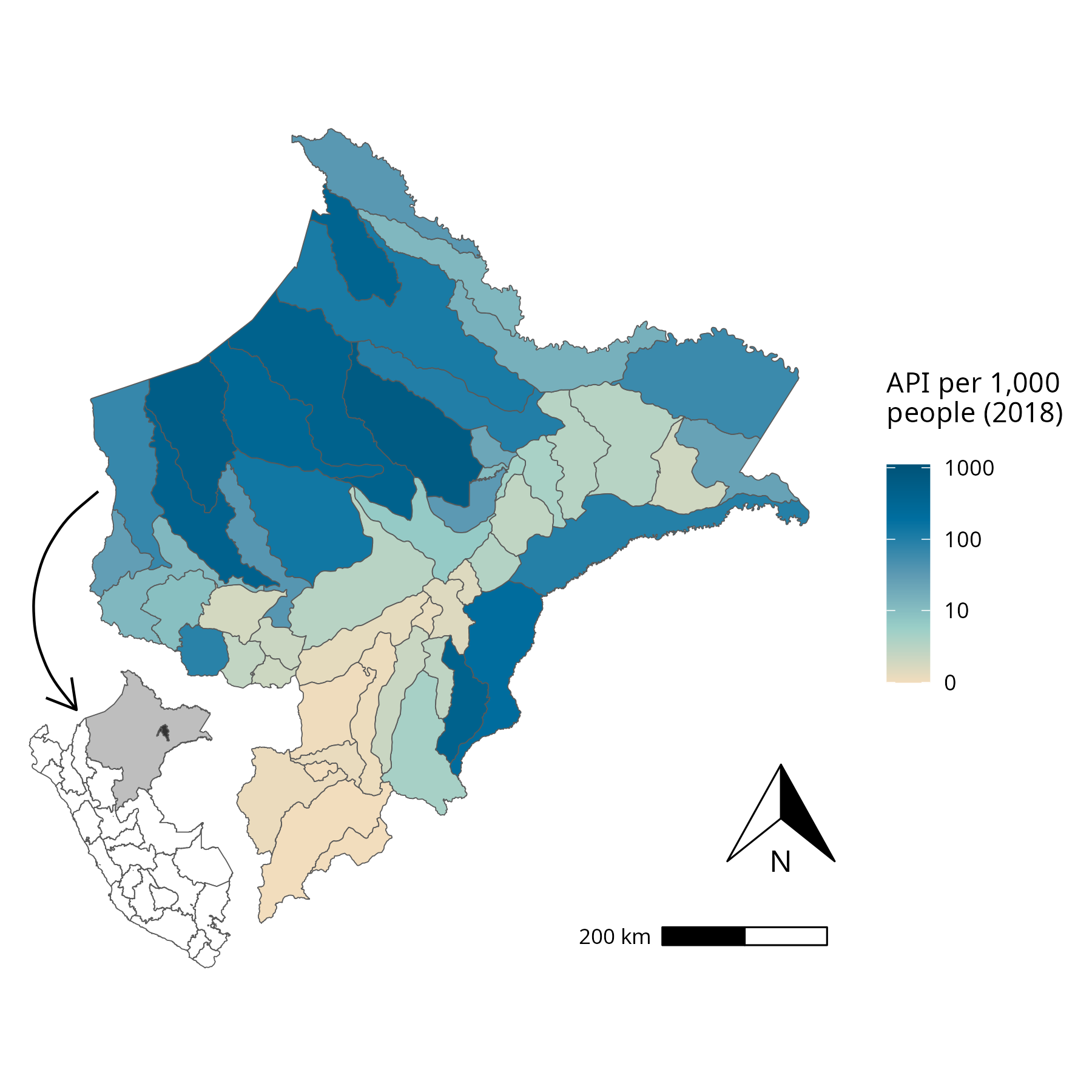


## **Supplementary Figure 2.** Sampling strategy. Flowchart of the selection process of (a) Stage 1: Health facilities selection, (b) Stage 2: Villages selection and (c) Stage 3: Households and individuals selection.


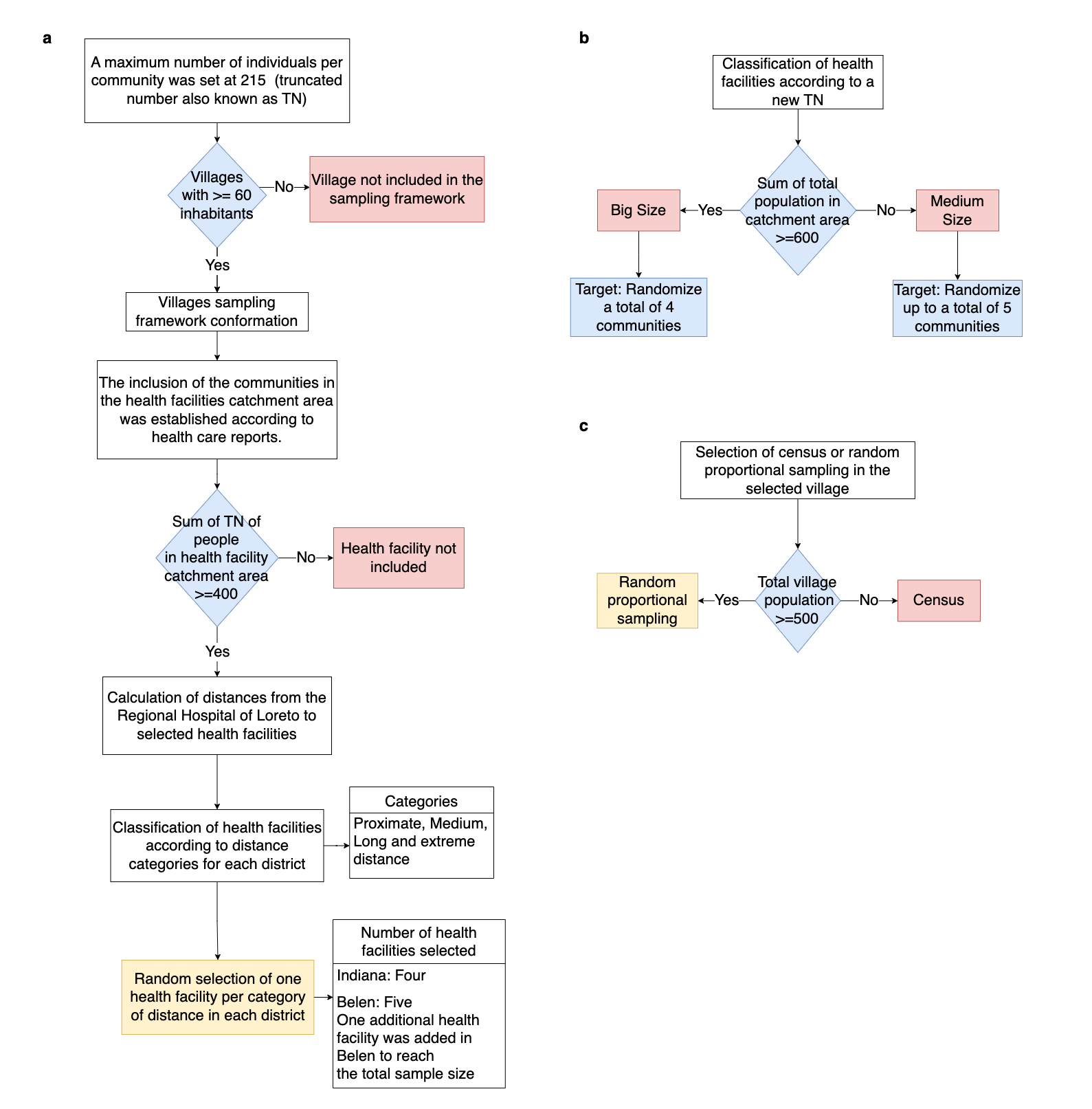


**Supplementary Figure 3.** Data management process. Flow of cleaning and debugging of collected data based on criteria such as duplicate records, erroneous information, missing information, inconsistency in the data, as well as absence of informed consent.

**
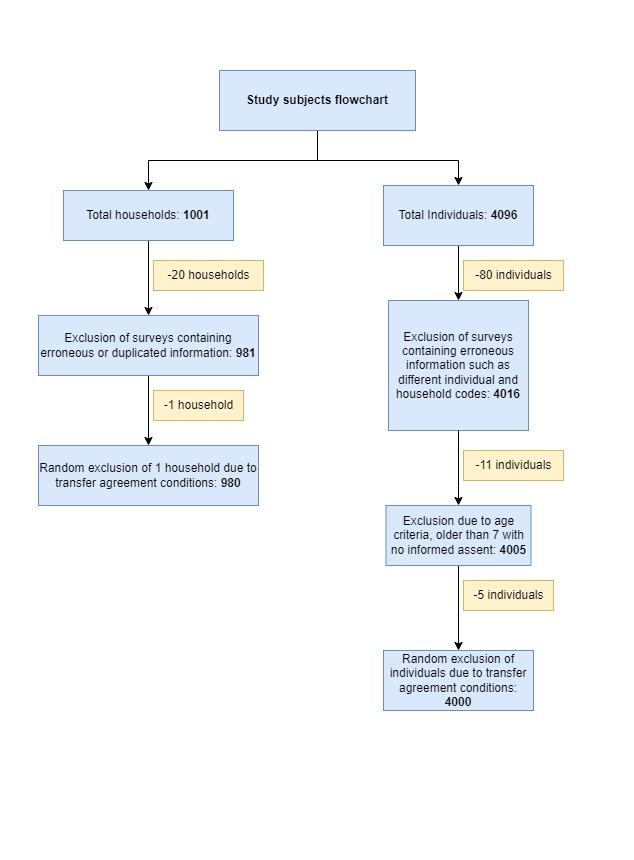
**

**Supplementary Figure 4.** Spatial analysis by village and district. Stacked bar plot showing the percentage of households classified as hot spots, cold spots or non-significant malaria exposure in each village for (a) *P. falciparum* in Belen, (b) *P. vivax* in Belen, (c) *P. falciparum* in Indiana and (d) *P. vivax* in Indiana. The bar plots were generated with R software v.4.1.2 (R Development Core Team, R Foundation for Statistical Computing, Austria. [http://www.R-project.org/](http://www.r-project.org/)).


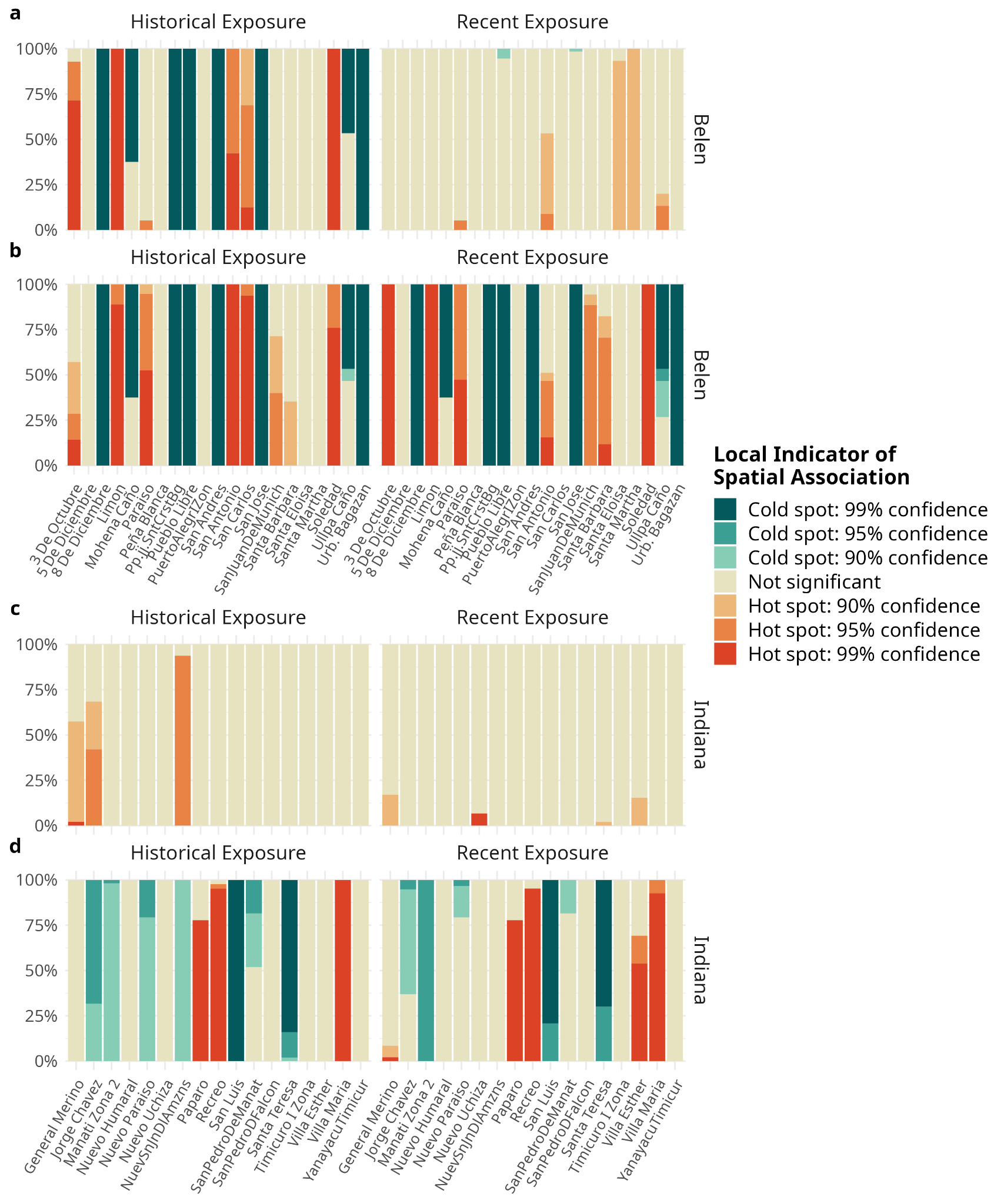

Supplement: Supplementary file 1 — Supplementary Information. [file 41598_2024_52239_MOESM1_ESM.docx]
